# A Critical Role of TET1/2 Proteins in Cell-Cycle Progression of Trophoblast Stem Cells

**DOI:** 10.1016/j.stemcr.2018.02.014

**Published:** 2018-03-22

**Authors:** Stephanie Chrysanthou, Claire E. Senner, Laura Woods, Elena Fineberg, Hanneke Okkenhaug, Sarah Burge, Vicente Perez-Garcia, Myriam Hemberger

**Affiliations:** 1 Epigenetics Programme, The Babraham Institute, Babraham Research Campus, Cambridge CB22 3AT, UK; 2 Centre for Trophoblast Research, University of Cambridge, Downing Street, Cambridge CB2 3EG, UK; 3 Imaging Facility, The Babraham Institute, Babraham Research Campus, Cambridge CB22 3AT, UK

**Keywords:** TET proteins, trophoblast stem cells, cell cycle, endoreduplication, self-renewal, mitosis, trophoblast giant cells, differentiation

## Abstract

The ten-eleven translocation (TET) proteins are well known for their role in maintaining naive pluripotency of embryonic stem cells. Here, we demonstrate that, jointly, TET1 and TET2 also safeguard the self-renewal potential of trophoblast stem cells (TSCs) and have partially redundant roles in maintaining the epithelial integrity of TSCs. For the more abundantly expressed TET1, we show that this is achieved by binding to critical epithelial genes, notably E-cadherin, which becomes hyper-methylated and downregulated in the absence of TET1. The epithelial-to-mesenchymal transition phenotype of mutant TSCs is accompanied by centrosome duplication and separation defects. Moreover, we identify a role of TET1 in maintaining cyclin B1 stability, thereby acting as facilitator of mitotic cell-cycle progression. As a result, *Tet1/2* mutant TSCs are prone to undergo endoreduplicative cell cycles leading to the formation of polyploid trophoblast giant cells. Taken together, our data reveal essential functions of TET proteins in the trophoblast lineage.

## Highlights

• TET1 and TET2 are critical for trophoblast stem cell (TSC) maintenance
• TET1 and TET2 safeguard TSC integrity by driving expression of epithelial genes
• TET1/2 null TSCs exhibit cell-cycle defects and become polyploid
• TET1 binds to and helps stabilize cyclin B1, thereby ensuring G2/M progression

## Graphical Abstract

**Figure:**
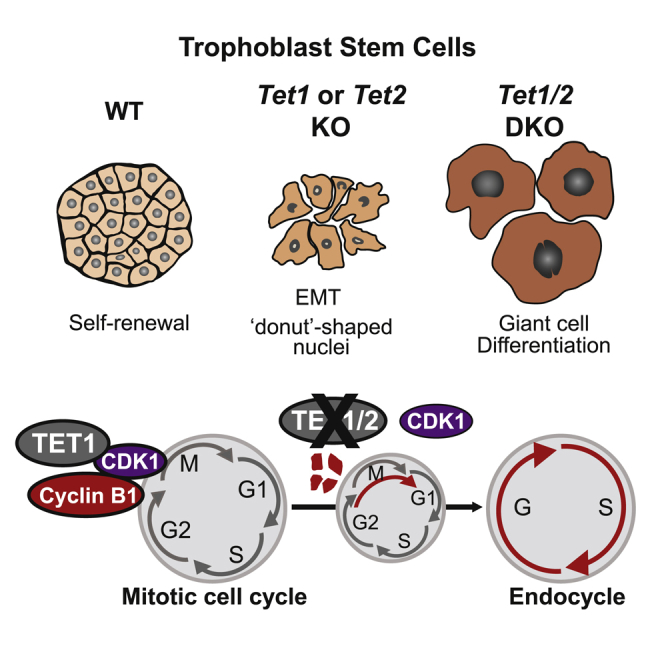

## Teaser

TET proteins are well known for their role in pluripotency. Here, Hemberger and colleagues show that TET1 and TET2 are also critical for maintaining the epithelial integrity of trophoblast stem cells. TET1/2 ensure mitotic cell-cycle progression by stabilizing cyclin B1 and by regulating centrosome organization. These insights reveal the importance of TET proteins beyond their role in epigenome remodeling.

## Introduction

Ten-eleven translocation (TET) proteins are epigenetic modifiers that catalyze the oxidation of 5-methylcytosine (5mC) into 5-hydroxymethylcytosine (5hmC) (He et al., 2011, Ito et al., 2011, Tahiliani et al., 2009). This reaction is the initial step in a series of events that ultimately leads to DNA demethylation. As such, the TET protein family (TET1, TET2, and TET3) has gained much attention in the field of epigenomic reprogramming during development and in stem cells (Amouroux et al., 2016, Dawlaty et al., 2014, Gu et al., 2011, Koh et al., 2011). Indeed, naive embryonic stem cells (ESCs) are enriched in 5hmC, but this epigenetic modification is rapidly lost as they differentiate. In pluripotent ESCs, TET1 and TET2 are the main proteins involved in the production of 5hmC (Koh et al., 2011). Accordingly, both genes are abruptly downregulated with differentiation (Costa et al., 2013, Koh et al., 2011). Moreover, *Tet1/2* knockout (KO) ESCs are depleted for 5hmC and are prone to differentiate (Dawlaty et al., 2013, Ito et al., 2010, Koh et al., 2011), indicating a direct functional role of these factors in ESC maintenance. In contrast, TET3 shows the opposite expression profile, as it is expressed at low levels in pluripotent ESCs but is upregulated upon differentiation (Koh et al., 2011). The essential role of TET proteins has also been demonstrated during early embryonic development as *Tet1/2/3* triple mutant embryos exhibit gastrulation defects and are embryonic lethal before mid-gestation (Dai et al., 2016).

Trophoblast stem cells (TSCs) can be viewed as the developmental counterpart of ESCs. Like ESCs, they can be derived from the blastocyst-stage mouse embryo, but they originate from the outer trophectoderm layer that is committed toward the trophoblast lineage, which ultimately gives rise to the major cell types of the placenta (Tanaka et al., 1998). TSCs can be maintained as a self-renewing stem cell population in culture, and they retain their entire differentiation repertoire *in vitro*, as well as *in vivo* when reintroduced into chimeras (Latos and Hemberger, 2016). This includes the unique ability of trophoblast to differentiate into giant cells through repeated rounds of endoreduplication, resulting in cells with a DNA content of up to 1,000N (Hemberger, 2008). While endoreduplication happens physiologically as part of the trophoblast giant cell (TGC) differentiation program, it can also be induced by depleting important cell-cycle proteins, particularly those which are part of the mitotic apparatus (Ullah et al., 2009). For example, chemical inhibition of cyclin-dependent kinase 1 (CDK1) in TSCs triggers endoreduplication accompanied by TGC differentiation (Ullah et al., 2008). The CDK1/cyclin B1 complex is one of the prime drivers of mammalian mitosis; thus, once the complex is disturbed, via CDK1 inactivation or absence of cyclin B1 from the nucleus, mitosis cannot take place. In the absence of mitosis, two main scenarios are possible; either initiation of endocycles in cells that are programmed to endoreduplicate, such as TSCs, or apoptosis in cells that are not, such as ESCs (Ullah et al., 2008).

In this study, we demonstrate that TET1 and TET2 are jointly required to maintain the stem cell state of TSCs. TET1/2 deletion triggers the initiation of trophoblast differentiation, reflected by an altered gene expression profile, increased ploidy and epithelial-to-mesenchymal transition (EMT). Importantly, we show that TET proteins have a unique role in the trophoblast cell cycle. TET1/2 are required for normal centrosome separation and G2-M progression via stabilization of cyclin B1, thereby enabling the CDK1-cyclin B1 complex to form, which is required to sustain the mitotic cell cycle in TSCs.

## Results

### TET1/2 Expression Is Associated with the Stem Cell State of TSCs

**Figure 1:**
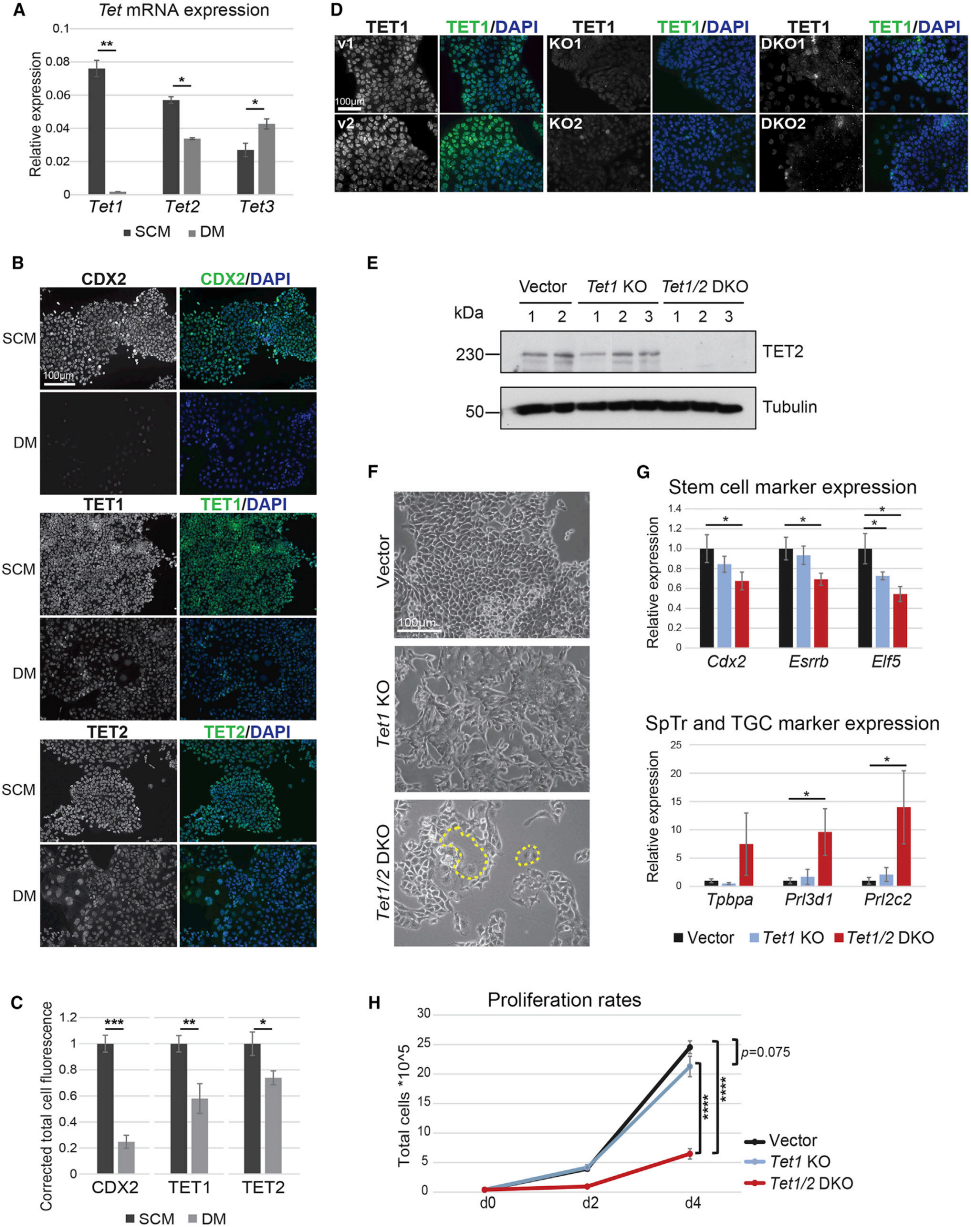
TET1 and TET2 Positively Correlate with the TSC State (A) qRT-PCR analysis of *Tet1,2,3* mRNA expression in TSCs cultured in stem cell media (SCM) or differentiation media (DM) over 3 days. Data are normalized against housekeeping genes *Sdha* and *Dynein*, and are displayed as mean ± SEM; ^∗^p < 0.05, ^∗∗^p < 0.01 (ANOVA with Holm-Bonferroni *post-hoc* test); n = 3 independent replicates each. (B) Immunofluorescence (IF) staining for CDX2, TET1, and TET2 in TSCs cultured in SCM and DM. Scale bar, 100 μm. (C) Quantification of total mean cell fluorescence of (B). ^∗^p < 0.05, ^∗∗^p < 0.01; ^∗∗∗^p < 0.001 (unpaired, one-sided t test). Measurements are of ≥100 cells each. (D) IF staining for TET1 in independent vector (v) control and *Tet1* KO and *Tet1/2* DKO clones. Scale bar, 100 μm. (E) Western blot for TET2 on vector control, *Tet1* KO and *Tet1/2* DKO TSCs. Tubulin was used as loading control. (F) Phase contrast images of vector control, *Tet1* KO and *Tet1/2* DKO clones grown in SCM. Yellow dotted lines indicate enlarged giant cell-like cells. Scale bar, 100 μm. (G) qRT-PCR analysis of TSCs and differentiation markers in vector control (set to 1) and mutant clones. SpTr, spongiotrophoblast; TGC, trophoblast giant cells. Data are mean ± SEM; ^∗^p < 0.05 (ANOVA with Holm-Bonferroni *post-hoc* test); n = 4 clones as independent replicates each. (H) Analysis of proliferation rates over a 4-day period. ^∗∗∗∗^p < 0.0001 (two-way ANOVA with Holm-Sidak's multiple comparisons test); n = 5 independent replicates each. See also Figures S1–S3.

Since TET proteins have been implicated in ESC self-renewal and pluripotency, we asked whether they might have similar functions in maintaining the stem cell state of TSCs. We confirmed that all three genes are expressed in TSCs, albeit *Tet1* and *Tet2* at much lower levels compared with ESCs (Figure S1A). Nonetheless, by assessing TSCs grown in stem cell conditions (stem cell media [SCM]) and after 3 days of differentiation (differentiation media [DM]), it was evident that *Tet1* and, to a lesser extent, *Tet2* mRNA levels were significantly higher in TSCs than in differentiated trophoblast cells, whereas *Tet3* was upregulated with trophoblast differentiation (Figure 1A). We further confirmed the downregulation of TET1 and TET2 with TSC differentiation on the protein level by immunofluorescence (IF) staining (Figures 1B and 1C). Selective withdrawal of either of the two growth factor requirements of TSCs, i.e., fibroblast growth factor (FGF) or the transforming growth factor β component commonly provided as fibroblast-conditioned medium, indicated that expression of *Tet1* as well as *Tet2* predominantly depended on FGF signaling (Figure S1B). Collectively, these data showed that akin to the situation in ESCs, TET1 and TET2 expression levels positively correlate with the stem cell state of TSCs.

### *Tet1/2* DKO TSCs Exhibit Diminished Stem Cell Potential

To define the role of TET1 and TET2 in maintaining the TSC state and their specific functions in the trophoblast compartment, we established *Tet1* single (KO) and *Tet1/2* double knockout (DKO) TSC lines by CRISPR/Cas9-mediated gene ablation using three different guideRNAs per gene to rule out prominent off-target effects. KO and DKO clones were confirmed by genotyping PCRs, Sanger sequencing, IF staining, and western blot (Figures 1D and 1E). They were also functionally validated based on global levels of 5hmC, which was significantly reduced in mutant clones (Figures S2A and S2B).

Morphologically, *Tet1* KO and *Tet1/2* DKO cells appeared more loosely organized and did not form the tight epithelial colonies characteristic of control TSCs (Figure 1F). Comparatively, however, the *Tet1/2* DKO TSCs exhibited a far more pronounced phenotype and contained a significant fraction of cells that were larger in size and exhibited morphological characteristics of TGCs (Figures 1F and S2C). To tease apart any additional effects incurred by TET2, we therefore also established *Tet2* single KO TSCs (Figure S2D). They appeared morphologically similar to *Tet1* single KOs, insofar as the cells were more loosely organized and lacked the sharp epithelial colony boundaries characteristic of TSCs (Figures S2E and S3), while not displaying as many markedly differentiated TGC-like cells as the DKOs. In line with these morphological observations, the expression levels of TSC markers were mildly reduced in *Tet1* and *Tet2* single KOs, but this decrease was more pronounced in the DKO cells (Figures 1G and S2F). Conversely, markers of spongiotrophoblast and TGCs were upregulated in the *Tet1/2* DKO TSCs, as expected from their appearance (Figure 1G). Accordingly, *Tet1/2* DKO clones also exhibited significantly slower proliferation rates (Figure 1H). A differentiation time course corroborated these general observations, with TGC differentiation generally accelerated in KO/DKO cells compared with vector control cells, while syncytiotrophoblast formation was delayed (Figures S2G and S3). Overall, these data indicated that both proteins may have partially redundant roles in maintaining the epithelial integrity of TSCs, whereas combined absence of TET1 and TET2 interferes more acutely with TSC maintenance.

### *Tet1/2* Null TSCs undergo EMT

**Figure 2:**
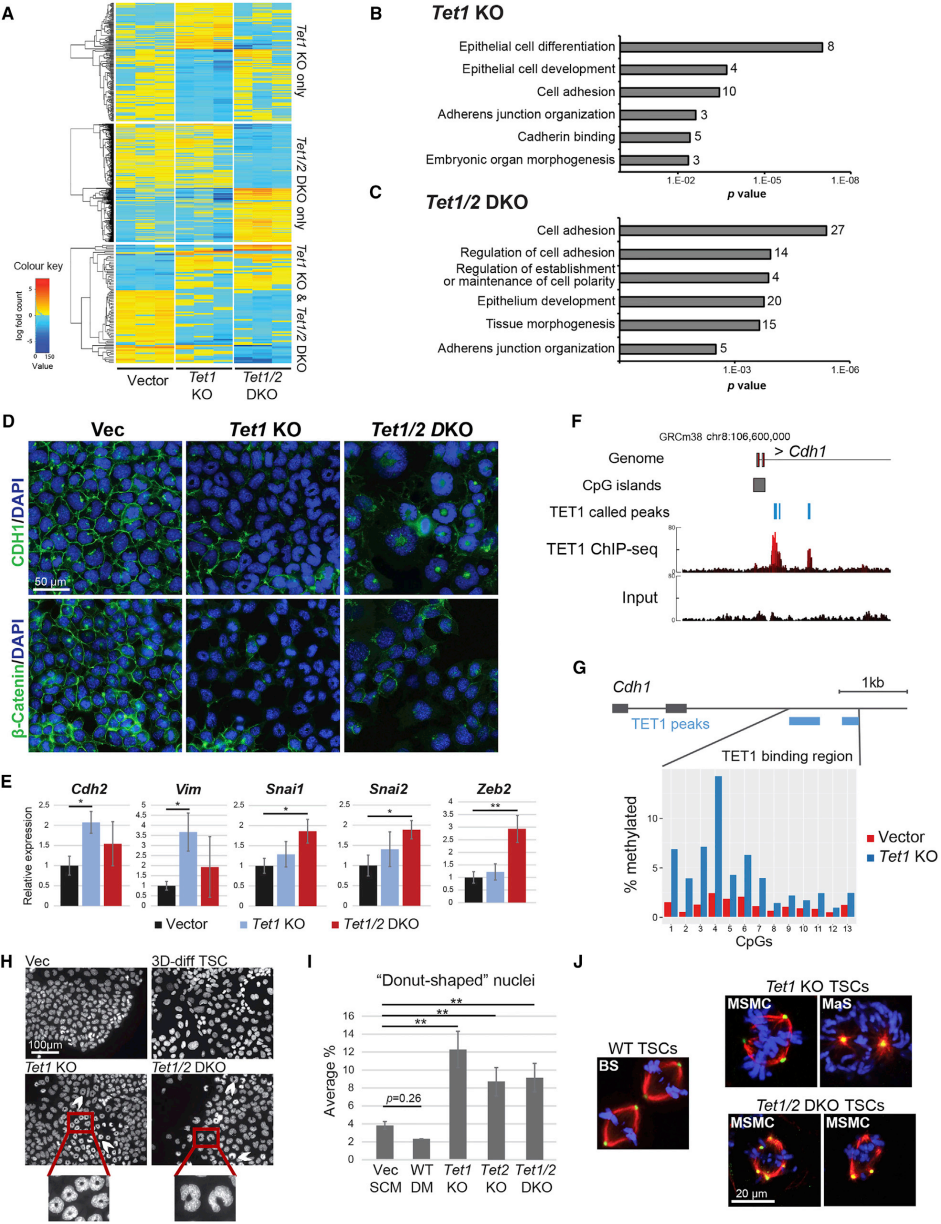
*Tet1* and *Tet1/2* Depletion Induces EMT and Centrosome Defects (A) Heatmap of differentially expressed genes between vector, *Tet1* KO, and *Tet1/2* DKO clones. n = 3 independent clones each. (B and C) Gene ontology term enrichment analysis for differentially expressed genes in *Tet1* KO cells (B) and in *Tet1/2* DKO cells (C). The numbers of genes in each enrichment term are also given. (D) IF staining for E-cadherin (CDH1) and β-catenin. Scale bar, 50 μm. (E) qRT-PCR expression analysis of mesenchymal markers. Data are mean ± SEM; ^∗^p < 0.05, ^∗∗^p < 0.01 (ANOVA with Holm-Bonferroni *post-hoc* test); n = 4 clones as independent replicates each. (F) TET1 ChIP-seq data displayed on a genome browser view for the *Cdh1* locus. Called peaks and actual peaks are displayed. (G) Bisulfite sequencing analysis across the TET1-bound region of the *Cdh1* locus, encompassing 13 individual CpG's, on vector control and *Tet1* KO TSCs. (H) DAPI-stained nuclei of vector, *Tet1* KO, and *Tet1/2* DKO clones grown in SCM, and 3-day differentiated WT TSCs. The white arrows point at ring- (donut) or crescent-shaped nuclei. Scale bar, 100 μm. (I) Unbiased quantification of donut-shaped nuclei by ImageJ analysis. WT DM are 3-day differentiated WT TSCs as shown in (H). ^∗∗^p < 0.01 (two-way ANOVA with Bonferroni *post-hoc* test). Measurements are of >400 cells each. (J) IF staining for α-tubulin (red), γ-tubulin (green), and chromatin (blue) in WT TSCs, *Tet1* KO, and *Tet1/2* DKO TSCs. Confocal images of representative spindle structures, including bipolar spindles (BS) in WT TSCs, multiastral spindles with multiple centrosomes (MSMC) resulting in misaligned chromosomes, and monoastral spindles (MaS), are shown. Scale bar, 20 μm. See also Figure S4.

To gain more detailed insights into the molecular changes caused by loss of TET1 and TET2, we performed global expression profiling by RNA-seq on the *Tet1* KO, *Tet1/2* DKO, and vector control cells. This analysis identified 210 and 349 genes that were differentially expressed in the *Tet1* KO and *Tet1/2* DKO clones, respectively (Figure 2A; Table S1). Of these, 25 genes were commonly de-regulated between the *Tet1* KO and *Tet1/2* DKO clones (Figure S4A), which included, for example, *Nr0b1*, a key TSC gene that is acutely linked to the stem cell state (Latos et al., 2015a). Using the differentially expressed genes for gene ontology (GO) analysis revealed that both the *Tet1* KO and *Tet1/2* DKO gene sets were significantly enriched in pathways related to epithelial integrity, cell polarity, and cell adhesion (Figures 2B and 2C). This corroborated our notion of a critical function of TET proteins specifically in maintaining the epithelial character of TSCs.

Loss of cell-cell adhesion is a defining characteristic of EMT, a process linked to the onset of trophoblast differentiation and the acquisition of invasive characteristics (Parast et al., 2001). A hallmark of EMT is the downregulation of E-cadherin (CDH1), an epithelial marker, from the cell surface. IF staining showed that membrane localization of CDH1 was indeed disrupted in *Tet1* KO, *Tet2* KO, and *Tet1/2* DKO clones (Figures 2D and S2E). β-Catenin, a ligand of the cytoplasmic part of E-cadherin, was also absent from the cell membrane of mutant cells (Figure 2D), confirming an *adherens* junction defect concordant with the morphological appearance of mutant TSCs (Figures 1F and S3B). To further corroborate these results, we analyzed a collection of mesenchymal markers by qRT-PCR and found that N-cadherin (*Cdh2*) and vimentin (*Vim*) were upregulated in particular in the *Tet1* KO cells, while the EMT-promoting transcription factors *Snai1*, *Snai2*, and *Zeb2* were significantly upregulated in the *Tet1/2* DKO clones (Figure 2E).

### TSC-Specific TET1 Genome Occupancy Regulates Epithelial Genes

To pinpoint the role of TET proteins in EMT, we performed chromatin immunoprecipitation (ChIP) followed by sequencing (ChIP-seq) for TET1 as the most highly and most differentially expressed TET family member in TSCs. This analysis identified 6,331 TET1 peaks (Table S2) that were globally concentrated around transcriptional start sites (Figure S4B). Analysis with the Genomic Regions Enrichment of Annotations Tool (McLean et al., 2010) identified that these TET1-bound sites were significantly associated with genes involved in embryonic lethality, and specifically placental development and TGC differentiation (Figure S4C), indicating a highly TSC-specific binding profile of TET1. When selecting those genes with promoter-associated TET1 peaks for GO analysis, the annotation categories “cell cycle” and “cell-cell *adherens* junctions” were among the top most significantly enriched terms (p = 2.78 × 10^−23^ and p = 1.97 × 10^−17^, respectively). Overall, expression levels of these genes were positively correlated with TET1 binding, as mean expression was higher in wild-type (WT) than in *Tet1* KO and *Tet1/2* DKO TSCs, although this effect was fairly mild (Figure S4D). This collection of TET1-bound EMT genes included the *Cdh1* locus itself (Figure 2F). Finally, we performed bisulfite sequencing of the *Cdh1* locus in WT and *Tet1* KO cells and found that DNA methylation levels are increased across the TET1-bound region in the absence of TET1 (Figure 2G).

Collectively, these results indicated that TET1 is necessary to maintain the epithelial character of trophoblast through direct regulation of critical EMT gene loci.

### Altered Nuclear Morphology of *Tet1* KO and *Tet1/2* DKO TSCs

During inspection of immunostained TSCs we noted that a substantial proportion of mutant cells exhibited “crescent”- or “donut”-shaped nuclei (i.e., nuclei with a central hole devoid of chromatin; Figure 2H), a phenotype that has been associated with defects in centrosome separation (Verstraeten et al., 2011). By contrast, this donut shape was far less frequently observed in WT TSCs, both in the stem cell state or after differentiation (Figure 2I). Treatment of TSCs with Monastrol, an inhibitor of the motor protein kinesin EG5, which is important for spindle bipolarity (Kapitein et al., 2005), recapitulated this nuclear phenotype, thus confirming a centrosome separation defect in *Tet1* KO and *Tet1/2* DKO TSCs (Figure S4E). IF staining for α-tubulin and γ-tubulin to examine spindle fibers and centrosomes, respectively, revealed that the donut-shaped mutant cells indeed exhibited centrally located centrosomes resulting in the formation of monoastral spindles (Kapoor et al., 2000), very similar to Monastrol-treated TSCs (Figures 2J and S4F). In addition, some mitotic *Tet1* and *Tet1/2* DKO cells appeared to have multiple centrosomes, causing multiastral fibers and chromosome misalignment (Figure 2J). Collectively, these data demonstrated that centrosome duplication and/or separation was defective in *Tet1*- and *Tet1/2*-depleted TSCs. Since centrosomes are important for the establishment of cell polarity, this nuclear rearrangement may also contribute to the EMT phenotype of *Tet* mutant TSCs (Agircan et al., 2014).

### *Tet1/2* DKO TSCs Exhibit Cell-Cycle Defects and Polyploidy

**Figure 3:**
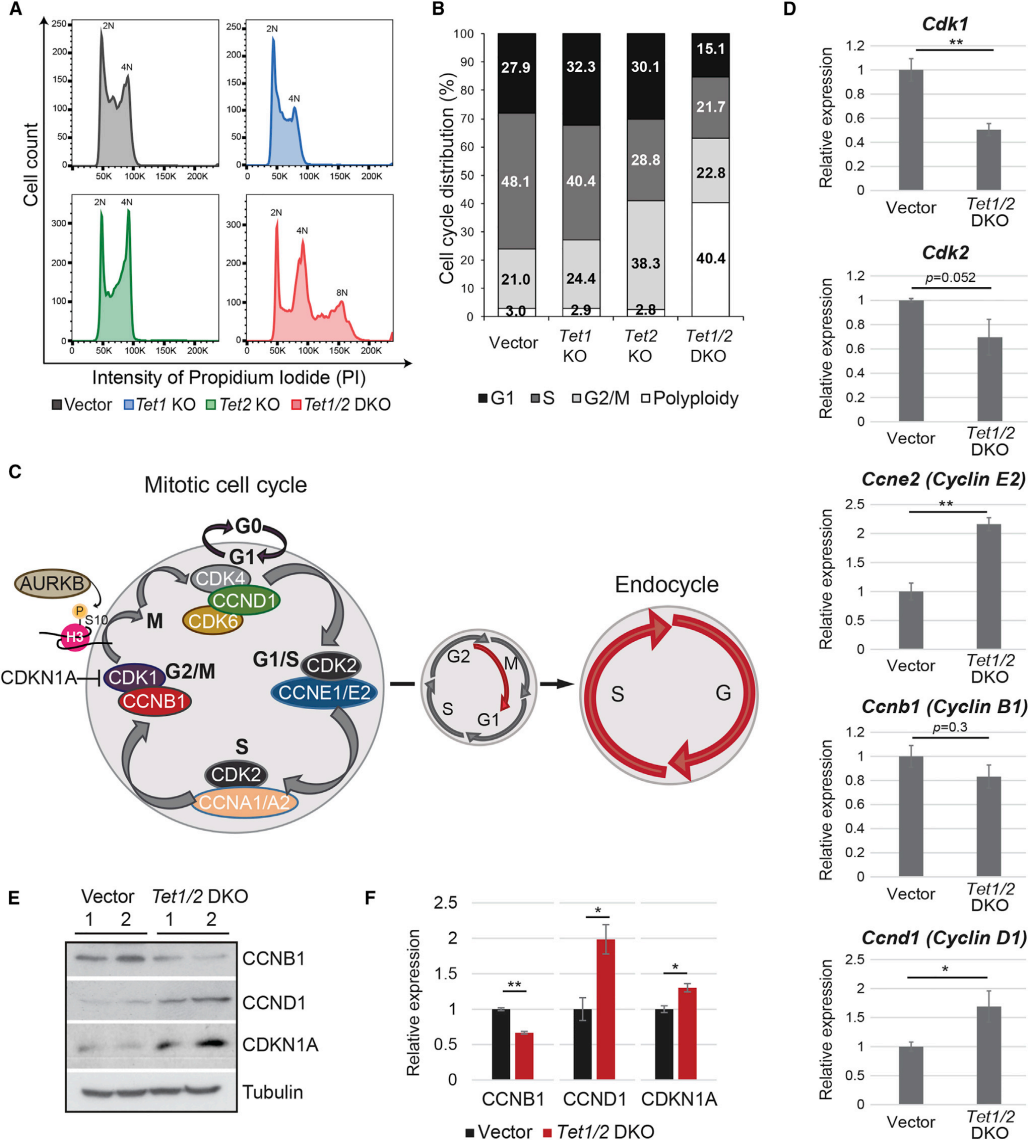
*Tet1/2* DKO Clones Undergo Endoreduplication (A) Cell-cycle distribution determined by propidium iodide (PI) staining of vector, *Tet1* KO, *Tet2* KO, and *Tet1/2* DKO cells. 2N DNA content indicates cells in G1 phase. 4N indicates cells in either G2 or M phase. >4N peaks denote polyploid cells. Graphs are representative of three independent experiments. (B) Quantification of data in (A) (C) Diagram showing the distinct cyclin-CDK complexes governing cell-cycle progression and the transition from a mitotic to an endoreduplicative cell cycle. (D) Expression analysis of cell-cycle components by qRT-PCR. Data are mean ± SEM; ^∗^p < 0.05, ^∗∗^p < 0.01 (ANOVA with Holm-Bonferroni *post-hoc* test); n = 3 independent replicates each. (E) Western blot analysis of cyclin B1 (CCNB1), cyclin D1 (CCND1), and CDKN1A (also known as P21) levels. Blots shown are representative of three independent replicates. Tubulin was used as loading control. (F) Quantification of protein band intensities of western blots in (E). Data are normalized against tubulin relative to vector control. ^∗^p < 0.05, ^∗∗^p < 0.01 (unpaired t test); n = 3 independent replicates.

Mutant cell morphology and the specific centrosome separation defects prompted us to investigate mitotic progression in KO and DKO cells more closely. Cell-cycle analysis by flow cytometry revealed that *Tet1/2* DKO cells, unlike *Tet1* and *Tet2* KO and vector cells, had undergone at least one endocycle, as evident by a broader 4N peak and a distinct 8N cell population, which constituted 40% of the total population (Figures 3A and 3B).

In normal mitotic cells, cyclins and CDKs govern faithful cell-cycle progression (Figure 3C). Endoreduplication is the result of mitosis bypass, such that DNA synthesis and G phase follow each other in the absence of cytokinesis. Hence, we first analyzed expression of a variety of cyclins and CDKs in *Tet1/2* DKO clones versus vector clones by qRT-PCR (Figure 3D). *Cdk1* expression was significantly decreased, whereas *Cyclin D1* (*Ccnd1*) and *Cyclin E2* (*Ccne2*) were upregulated upon *Tet1/2* deletion. E-Cyclins are particularly indicative of endocycling TGCs, thus confirming the above observations (Parisi et al., 2003). Although *Cyclin B1* (*Ccnb1*) and *Cdk2* expression remained unchanged, cyclin B1 protein levels were significantly lower in *Tet1/2* mutant cells, indicating a post-transcriptional effect of TET1/2 depletion on cyclin B1 stability (Figures 3E and 3F). In addition, cyclin D1 and cyclin-dependent kinase inhibitor 1A ([CDKN1A], also known as P21) protein levels were significantly increased in *Tet1/2* DKO cells (Figures 3E and 3F). The upregulation of CDKN1A in combination with decreased cyclin B1 suggested a potential defect in the mitotic machinery of *Tet1/2* DKO cells, specifically at the level of CDK1 activity, as CDK1 can be inhibited by CDKN1A activity and rendered inactive in the absence of cyclin B1 (Castedo et al., 2002).

### TET1/2 Are Required for Normal G2 to M Progression

**Figure 4:**
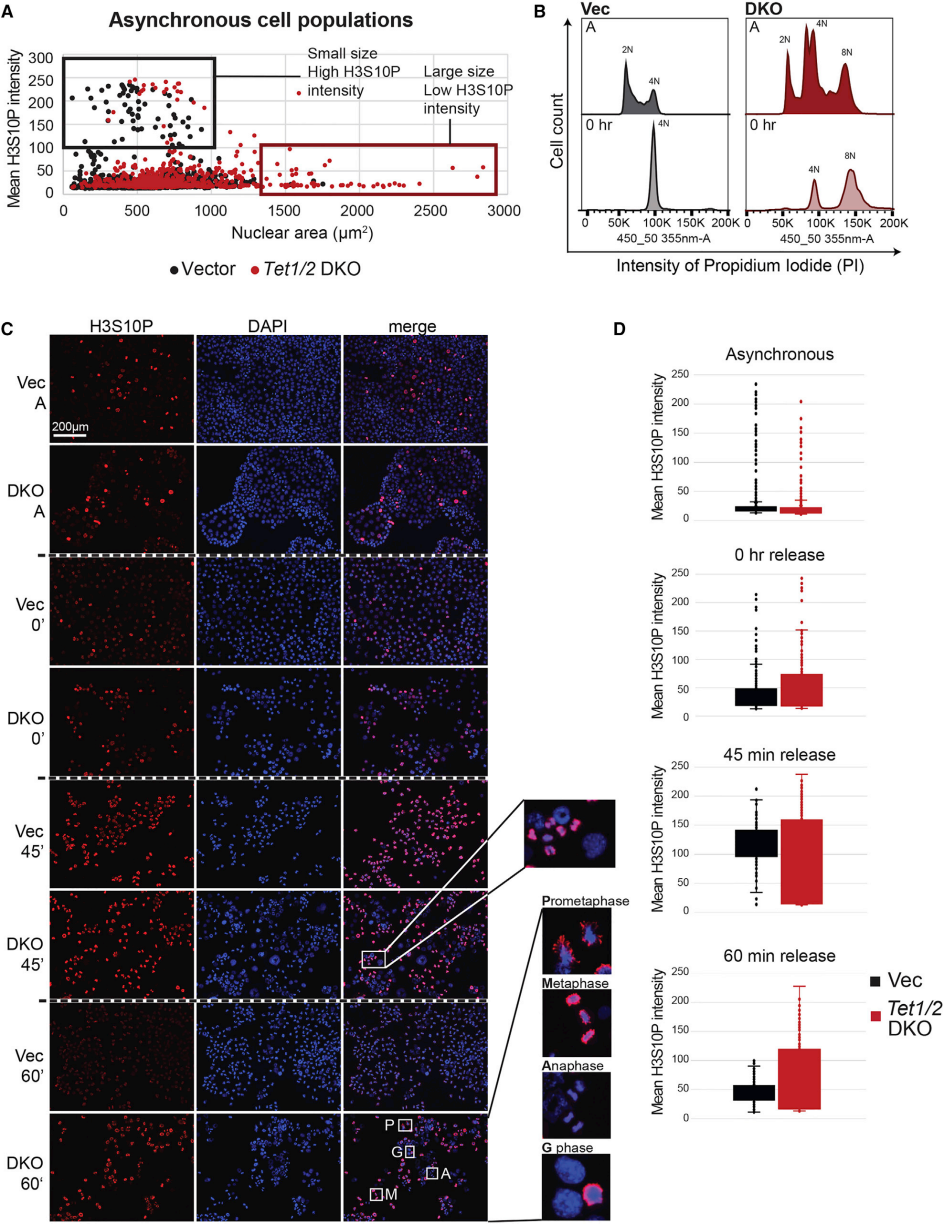
TET1/2 Are Required for Normal G2/M Progression (A) Scatterplot showing the mean H3S10P intensity plotted against nuclear surface area in asynchronous vector and *Tet1/2* DKO cells. (B) Cell-cycle distribution of asynchronous (“A”) and G2/M arrested (0 hr) vector and *Tet1/2* DKO cells. The cells were stained with PI and analyzed by flow cytometry on a linear scale. (C) IF staining for H3S10P in asynchronous and synchronized vector and *Tet1/2* DKO cells. Cells were arrested at G2/M phase with RO3306, 0 hr, and released with fresh medium for 45 and 60 min. DAPI staining (blue) was used to identify live nuclei. Scale bar, 200 μm. (D) Quantification of mean H3S10P intensity in asynchronous, arrested (0 hr), and released vector and *Tet1/2* DKO cells. Measurements are of >150 cells each per time point and genotype.

The above results suggested that the propensity of *Tet1/2* DKO TSCs to enter the endoreduplicative cell cycle may be a consequence of a de-regulated mitotic machinery, specifically at the level of the G2/M players CDK1 and cyclin B1. Hence, we studied G2-M progression in more detail by staining for phosphorylated histone H3 serine 10 (H3S10P), a marker of chromosome condensation at late G2 phase. When plotting H3S10P intensity against cell size in asynchronous cell populations, it was clear that most of the large post-mitotic H3S10P-negative cells were comprised of *Tet1/2*-null cells, whereas the majority of cells with highest H3S10P intensity were small and almost exclusively made up of vector control cells (Figure 4A). These results corroborated our previous data on DKO cell size and proliferation differences. We then arrested cells at G2/M with the CDK1 inhibitor RO3306, followed by release at specific time points (Figures 4B and S5A). In vector control cells, H3S10P intensity peaked at 45 min post-release, indicating that most cells had progressed into the early stages of mitosis (Figures 4C and 4D). In contrast, many of the *Tet1/2* DKO cells stained negative for H3S10P at this time point, indicating that these cells were post-mitotic. At 60 min post-release, vector cells had progressed into anaphase as most of the H3S10P had disappeared. In the case of *Tet1/2* mutant cells, however, some H3S10P staining still persisted, implying that even the mitotic population progressed more slowly through G2 and/or M phase than control cells (Figures 4C and 4D). Moreover, Aurora kinase B (AURKB), the kinase that phosphorylates H3S10, was clearly downregulated in the released *Tet1/2* DKO cells compared with vector controls (Figures S5B and S5C).

Overall, these results showed that *Tet1/2* deletion resulted in a generally slower cell-cycle progression, specifically through G2-M, which is likely to favor the bypass of mitosis and initiation of endoreduplication in TSCs.

### TET1/2 Stabilize Cyclin B1

**Figure 5:**
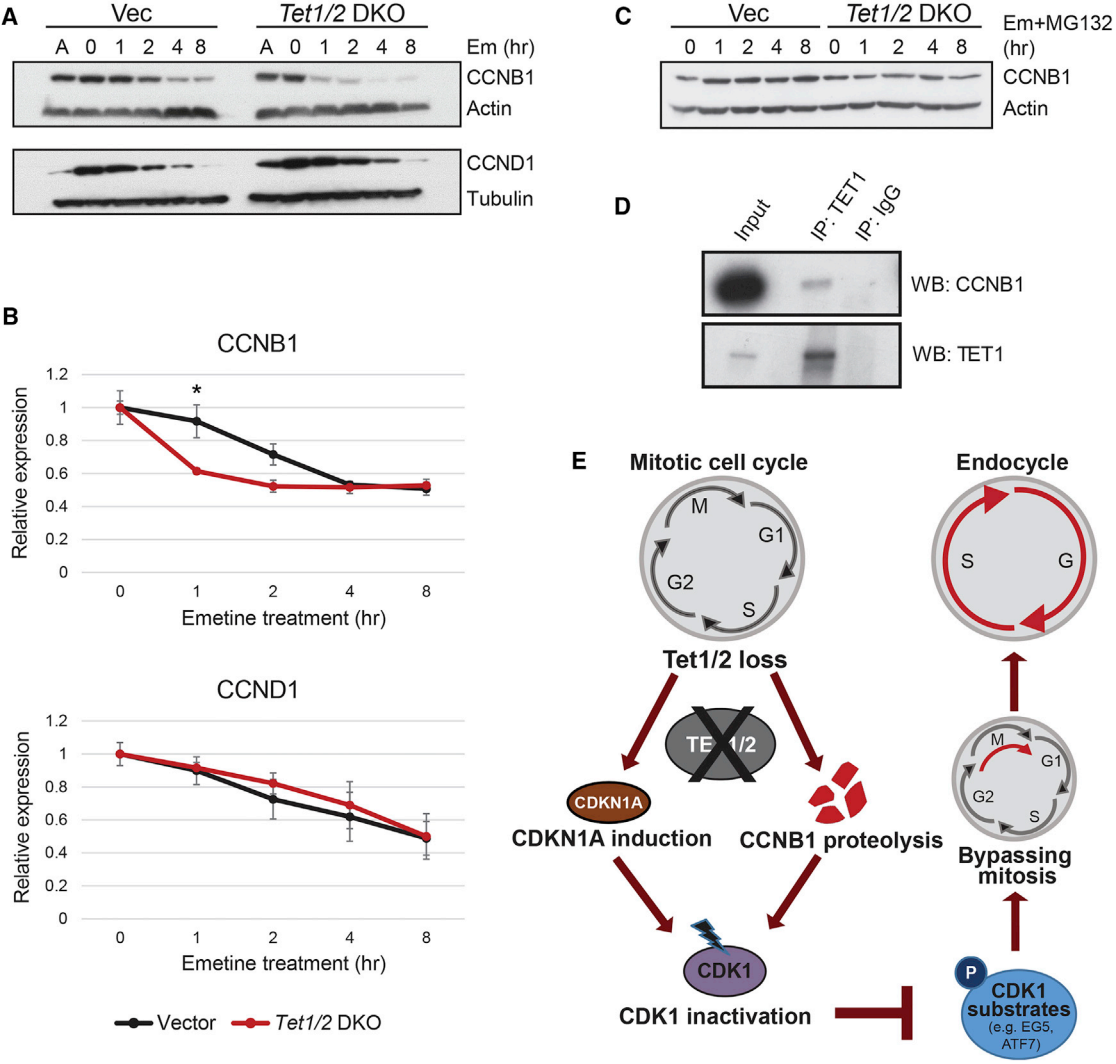
TET1/2 Stabilize Cyclin B1 (A) Western blots to determine cyclin B1 (CCNB1) and cyclin D1 (CCND1) stability in TSCs treated with the translation inhibitor emetine for the indicated time periods. (B) Quantification of band intensities (n = 4 independent replicates for cyclin B1 [CCNB1]; n = 2 independent replicates for cyclin D1 [CCND1]; ^∗^p < 0.05 [two-way ANOVA with Holm-Sidak's multiple comparisons test]. 0 hr value was set to 1. (C) Analysis of cyclin B1 (CCNB1) levels following combined translation and proteasome inhibition with emetine (Em) and MG132 over the indicated time points. (D) Western blot of TET1 co-immunoprecipitates from ESCs assessed for cyclin B1 (CCNB1) interaction. (E) Model of cell-cycle defects induced by loss of TET1/2 depletion in TSCs. TET1/2 depletion results in CDKN1A (P21) upregulation, as well as enhanced cyclin B1 (CCNB1) degradation, both rendering CDK1 inactive. CDK1 inactivation prevents phosphorylation of CDK1 substrates, such as ATF7, which promotes M-phase entry via stabilization of AURKB, and the motor protein EG5, which is involved in bipolar spindle formation. Collectively, a defective G2/M progression results in bypass of mitosis and entry into the endocycle. See also Figure S5.

In an attempt to explain how mitosis is bypassed in *Tet1/2* DKO cells, we examined the possibility of compromised cyclin B1 stability upon TET1/2 depletion, led by the significantly lower cyclin B1 protein levels in the *Tet1/2* DKO clones (Figures 3E and 3F). We arrested vector and *Tet1/2* DKO cells in G2/M phase to enrich for cyclin B1 protein, and then set up a protein stability assay by treating the cells with emetine, a translation inhibitor (Figure S5D). These data showed that cyclin B1 degraded much faster in the mutant cells than in controls, whereas other cyclins, notably cyclin D1, remained unchanged (Figures 5A and 5B). Proteasome inhibition with MG132 showed that the faster cyclin B1 degradation in *Tet1/2* DKO cells is proteasome dependent (Figures 5C and S5D). To further explain the mechanism by which TET proteins may be able to stabilize cyclin B1, we performed co-immunoprecipitation experiments, which revealed that cyclin B1 interacts with TET1 (Figure 5D). Collectively, these data suggest that TET1 binds to cyclin B1 and may help stabilize it, thereby ensuring G2/M progression in WT cells.

## Discussion

TET proteins have been extensively studied for their role in epigenetic reprogramming during development and in maintaining ESC pluripotency (Dawlaty et al., 2014, Ficz et al., 2011, Koh et al., 2011). Yet their role in the extra-embryonic lineage, and in particular in TSCs, has not been elucidated to date. Here, we show that TET1 and TET2 are critical for the maintenance of self-renewal potential and epithelial integrity of TSCs. Focusing on TET1 as the predominant TET family member in TSCs, we show that its genomic binding pattern is specific to the trophoblast compartment and includes a number of genes involved in epithelial integrity. In line with TET1's well-recognized epigenetic modifier activity, these loci appear to rely on TET1 binding to prevent the accumulation of repressive DNA methylation. Moreover, we uncover an important function of TET proteins in ensuring normal mitotic cell-cycle progression in trophoblast. Overall, these data underpin earlier evidence that suggested a role of the TET proteins, and specifically of TET1, in the placental trophoblast lineage (Dawlaty et al., 2011, Dawlaty et al., 2013, Khoueiry et al., 2017).

Interestingly, even though *Tet1/2* DKO cells exhibit a giant cell-like phenotype as indicated by their morphology, DNA content and increased giant cell marker expression, they did not seem to be functionally equivalent to WT TGCs. Thus, DKO cells did not acquire ploidy levels equivalent to *in vitro* differentiated TGCs, and the accumulation of ring- or crescent-shaped nuclei was only evident in mutant cells but was almost never observed in WT TSCs even upon differentiation. Thus, entering into the TGC path does not appear to be the result of a general differentiation-promoting mechanism that is triggered in the absence of TET1/2, but rather the direct consequence of impaired cell-cycle progression.

An intriguing phenotype of *Tet1* and *Tet1/2* mutant TSCs was the occurrence of centrosome abnormalities and defective spindle fiber formation. Specifically, *Tet1* and *Tet1/2* depletion resulted in mis-coordinated centrosome separation and progression of centriole duplication, evident by tripolar spindle formation arising from three centrioles, as well as multiastral spindles with multiple centrioles within individual mitotic mutant cells. The precise nature of these centrosome defects remains to be defined, insofar as centriole disengagement and/or centriole replicative growth may be faulty. In any case, since centrosome organization is closely linked to the establishment of cell polarity (Agircan et al., 2014), it is highly likely that the EMT phenotype that we commonly observed in the KO and DKO cells is tied to these nuclear rearrangements.

While the above phenotypes are shared between single and compound mutant TSCs, KO and DKO cells are distinct as far as proliferation rates and DNA content are concerned. These data suggest partially redundant functions of both, TET1 and TET2 proteins in cell-cycle progression, a conclusion underpinned by similar observations during mammalian development (Dai et al., 2016, Dawlaty et al., 2013). This also implies that centrosome defects alone do not explain the slower proliferation rates and increased frequency of endoreduplication. A previous study showed that both TET1 and TET2 regulate the transcription of genes related to DNA replication (e.g., *Mcm2/3/4/5/6*) and cell-cycle progression (e.g., *Ccne1*, *Md2l1*, and *E2f2*) in neural stem cells (Shimozaki, 2017). We also observe TET1 enrichment at cell-cycle gene loci where it likely serves to maintain an open, active chromatin configuration, akin to our findings at the *Cdh1* locus. In addition, we show that TET1 physically interacts with cyclin B1, potentially stabilizing it. Thus, upon *Tet1/2* depletion cyclin B1 degrades much faster compared with vector control cells. The reduced stability of cyclin B1 explains its decreased levels in *Tet1/2* DKO cells. Lower cyclin B1 combined with increased CDKN1A abundance may interfere with normal CDK1 activation, a key requirement for timely G2/M progression (Figure 5E). CDK1 activation is also important to phosphorylate the motor protein EG5, triggering centrosome separation in late G2 phase (Smith et al., 2011). Indeed, inactivation of CDK1 in *Drosophila* converted mitotic wing disc cells into endoreduplicating cells with aberrant centriole duplication, giving rise to triple centriole configuration (Vidwans et al., 2003), a very similar phenotype to *Tet1/2* DKO TSCs. Interestingly, cancer cells are also often associated with aberrant centriole morphologies, indicating that our observations might give insight into defects underlying the mitotic instability of cancer cells.

Overall, we show that jointly, TET1 and TET2 safeguard the plasticity and self-renewal capacity of TSCs by maintaining epithelial integrity and preventing entry into the endoreduplicative cell cycle. Our data reveal a role of TET1/2 in cell-cycle progression, a function that may be exerted by multiple distinct mechanisms: canonical DNA binding of TET proteins helps maintain an active chromatin configuration at target loci and ensures adequate transcriptional output. In addition, we find that TET1/2 ablation is associated with a decrease in cyclin B1 levels, and suggest that TET1 may affect cyclin B1's stability by protein-protein interaction. Since, to the best of our current knowledge, the catalytic activity of TET proteins is solely targeted to nucleotide modifications, notably the oxidative conversion of 5mC to 5hmC and its downstream products, this function appears unrelated to epigenome modulation and expands our knowledge of the roles of TET's in key cellular processes.

## Experimental Procedures

### Stem Cell Culture

TSC lines were blastocyst-derived TS-Rs26, a kind gift of the Rossant lab, Toronto, Canada. TSCs were cultured in routine conditions (Senner et al., 2012, Tanaka et al., 1998). CRISPR-Cas9-mediated gene ablation was performed as described previously (Murray et al., 2016). Clones obtained were genotyped by PCR using primers spanning the targeted exon (Figure S2; Table S3); selected clones (five each) were further confirmed by Sanger sequencing, by IF (TET1), and by western blot (TET2). For G2/M arrest, RO3306 (CDK1 inhibitor, Calbiochem-Merck, 217699) was added to SCM at a final concentration of 8 μM. For inhibition of bipolar spindle formation, cells were treated with 100 μM monastrol (CamBioScience, CAY15044) for 20 hr. Translation inhibition was achieved with 10 μM emetine (Sigma, E2375). Ten μm of MG132 (Calbiochem, Merck, 474787) was used for proteasome inhibition.

### IF Staining

For staining of cultured cells, cells were grown on coverslips, fixed with 4% paraformaldehyde for 10 min and permeabilized with PBS, 0.1% Triton X-100 for 15 min, or fixed and permeabilized with 100% methanol for 10 min. Blocking was carried out with PBS, 0.1% Tween 20, 0.5% BSA (PBT/BSA), followed by antibody incubation for 60 min. Primary antibodies and dilutions (in PBT/BSA) were: E-cadherin (CDH1) 1:200 (BD Biosciences, 610181), β-catenin 1:400 (BD Biosciences, 610153), 5hmC 1:2,000 (Active Motif, 39769), TET1 1:750 (GeneTex, GTX125888), TET2 1:100 (Abcam, ab124297), CDX2 1:400 (BioGenex, MU392-UC), α-tubulin 1:1,000 (Abcam, ab6160), and γ-tubulin 1:250 (Santa Cruz, sc7396). Primary antibodies were detected with the appropriate secondary Alexa Fluor 488 or 568 (Thermo Fisher Scientific) antibodies. Nuclei were counter-stained with DAPI. Photographs were taken with an Olympus BX61 epifluorescence microscope or a Zeiss LSM 780 confocal microscope.

Nuclear morphology (shape) was analyzed in ImageJ. In brief, after application of a background filter separate images were created of nuclei and donut holes. Their segmentation allowed for the identification of donut-shaped nuclei by the presence of a hole. Data were processed in Excel.

### Cell-Cycle and Proliferation Analysis

Cells were fixed in 70% ethanol/PBS and stained for 30 min in PBS containing 0.1 mg/mL RNAse and 50 μg/mL propidium iodide. Analysis was on a BD LSR II flow cytometer using FlowJo software.

For proliferation assays, 40,000 cells were seeded and viable cell counts determined after 2 and 4 days using the Muse Count & Viability Assay Kit (Merck Millipore, MCH100102) and Muse Cell Analyzer (Merck Millipore). Two-way ANOVA test was used to calculate statistical significance. All image quantification analyses were performed with ImageJ.

### qRT-PCR Expression Analysis

Gene expression analysis was performed using a standard protocol (Murray et al., 2016). qPCR was performed using SYBR Green JumpStart Taq ReadyMix (Sigma, S4438) and intron-spanning primer pairs (Table S3) on a Bio-Rad CFX96 or CFX384 thermocycler. Ct values were normalized to housekeeping genes (*Sdha* and *Dynein*). Where appropriate, Student's t test or ANOVA was performed to calculate statistical significance of expression differences. For all qRT-PCR data, n specifies biological replicates performed with independently derived clones.

### Transcriptome Sequencing (RNA-Seq)

Total RNA was extracted using TRI Reagent (Sigma, T9424), followed by DNase treatment using the TURBO-DNA-free Kit (Life Technologies, AM1907). mRNA was isolated using the Dynabeads mRNA Purification Kit (Life Technologies, 61006) and processed into indexed, strand-specific libraries using the ScriptSeq v.2 RNA-Seq Library Preparation Kit (Epicentre, SSV21106). Libraries were quantified and assessed using the KAPA Library Quantification Kit (KAPA Biosystems, KK4824) and Bioanalyzer 2100 System (Agilent). Indexed libraries were sequenced with a 100 bp single-end protocol on an Illumina HiSeq 2500 sequencer.

### ChIP-Seq

ChIP-seq library generation was carried out as described previously (Latos et al., 2015b). A total of 150 μg chromatin and 5 μg TET1 antibody (GeneTex, GTX125888) were used for each ChIP. ChIP as well as Input libraries were generated in biological triplicates. Sequencing libraries were quantified as above by Bioanalyzer 2100 System (Agilent) and using the KAPA Library Quantification Kit (KAPA Biosystems, KK4824). Indexed libraries from ChIP and Input samples were sequenced with a 50 bp paired-end protocol on an Illumina HiSeq 2500 sequencer.

### Bisulfite Sequencing

DNA from vector control (WT) and *Tet1* KO cells was bisulfite-converted using the EpiTect Kit (QIAGEN, 59104). The *Cdh1* locus was amplified with KAPA HiFi Hotstart Uracil+ ReadyMix (Roche, KK2801) using the primers listed in Table S3, which included a sequencing adaptor on the forward and reverse primers. The product was amplified and indexed using KAPA HiFi HotStart ReadyMix (Roche, KK2602). Libraries were quantified as above by Bioanalyzer 2100 System (Agilent) and using the KAPA Library Quantification Kit (KAPA Biosystems, KK4824), and sequenced with a 150 bp paired-end protocol on a MiSeq sequencer (Illumina), with 10% PhIX spike-in.

### Bioinformatic Analysis

RNA-seq raw fastq data were trimmed with trim-galore, using default parameters, and mapped to the *Mus musculus* GRCm38 genome assembly using TopHat v.2.0.12. Data were quantitated using the RNA-seq quantitation pipeline in Seqmonk software (www.bioinformatics.babraham.ac.uk), producing log2 reads per million reads of input values. A differentially expressed gene list was compiled through EdgeR analysis (p < 0.05), and heatmaps were produced based on hierarchical clustering. GO analysis was carried out using the DAVID bioinformatic tool (Huang da et al., 2009).

ChIP-seq raw fastq data were trimmed with trim-galore and then mapped to the *Mus musculus* GRCm38 genome assembly using Bowtie v.2.0. Peaks were called from three replicates with MACS2 (Zhang et al., 2008) using default parameters.

Bisulfite sequencing data were mapped to the *Mus musculus* GRCm38 genome assembly and processed using Bismark (www.bioinformatics.babraham.ac.uk) to find the percent methylation for each CpG. CpGs with <30 reads were filtered out, leaving CpGs 2–5, and 8–16 of the amplified region displayed in Figure 2G.

### 5-hmC Quantification

For unbiased quantification of total 5hmC by mass spectrometry, cells were lysed, DNA prepared, and analyzed by mass spectrometry as described previously (Senner et al., 2012). For relative quantification by dot blot, genomic DNA dilutions at 2, 1, and 0.5 μg were denatured, spotted on Amersham Hybond-N+, Nytran supercharge membrane, and UV crosslinked. The membrane was incubated with anti-5hmC antibody (Active Motif, 39769) at 1:10,000 at 4°C overnight, followed by detection with horseradish peroxidase-conjugated secondary antibody and ECL Reagent (GE Healthcare, RPN2106).

### Protein Co-immunoprecipitation

TSCs were washed and collected in 3 mL ice-cold PBS supplemented with protease inhibitors (Roche, 1836170). Nuclear extract preparations and co-immunoprecpation were performed essentially as described previously (Latos et al., 2015b), using Protein G Dynabeads (Thermo Fisher Scientific, 10004D) crosslinked to antibodies. Western blot analysis of the IPs along with immunoglobulin G-IPs and input controls were performed as described.

### Western Blotting

Protein lysates and western blots were prepared according to routine protocols. Primary antibodies used were: cyclin D1 1:200 (Merck Millipore, cc-12), cyclin B1 1:500 (Neomarker, Thermo Scientific, MS-868), AURKB 1:1,000 (Cell Signaling Technology, 3094), P21 (CDKN1A) 1:500 (BD Biosciences, 556431), TET2 1:1,000 (Abcam, ab124297), tubulin 1:2,500 (Abcam, ab6160), actin 1:2,000 (Santa Cruz, sc7210), and HSP90 1:5,000 (BD Biosciences, 610418). For all western blot data, n specifies biological replicates.

## Author Contributions

S.C., C.E.S., and V.P.-G. performed the bulk of the experiments. L.W. and S.B. performed bioinformatic analyses of RNA-seq, ChIP-seq, and bisulfite-seq data. E.F. and H.O. performed image analyses. S.C., C.E.S., S.B., L.W., V.P.-G., and M.H. designed the experiments, performed data interpretation, and wrote the manuscript.

## Accession Numbers

All sequencing data (RNA-seq, ChIP-seq, and bisulfite sequencing of the *Cdh1* locus) have been deposited under GEO: GSE109545.

## Supplemental Information

Document S1. Figures S1–S5 and Table S3

Table S1. List of Genes De-regulated in Tet1 Single and Tet1/2 Double Knockout TSCs, Related to Figure 2

Table S2. TET1 ChIP-Seq Peaks in TSCs, Related to Figure 2

Document S2. Article plus Supplemental Information
