# Supplementary material for: A Critical Role of TET1/2 Proteins in Cell-Cycle Progression of Trophoblast Stem Cells
Source: Stem Cell Reports. 2018 Mar 22;10(4):1355–68. doi: 10.1016/j.stemcr.2018.02.014 (PMC5998911; doi:10.1016/j.stemcr.2018.02.014)
Supplement: Document S1. Figures S1–S5 and Table S3 [file mmc1.pdf]

Stem Cell Reports, Volume *10*

Supplemental Information

A Critical Role of TET1/2 Proteins in Cell-Cycle Progression of Trophoblast Stem Cells

Stephanie Chrysanthou, Claire E. Senner, Laura Woods, Elena Fineberg, Hanneke Okkenhaug, Sarah Burge, Vicente Perez-Garcia, and Myriam Hemberger

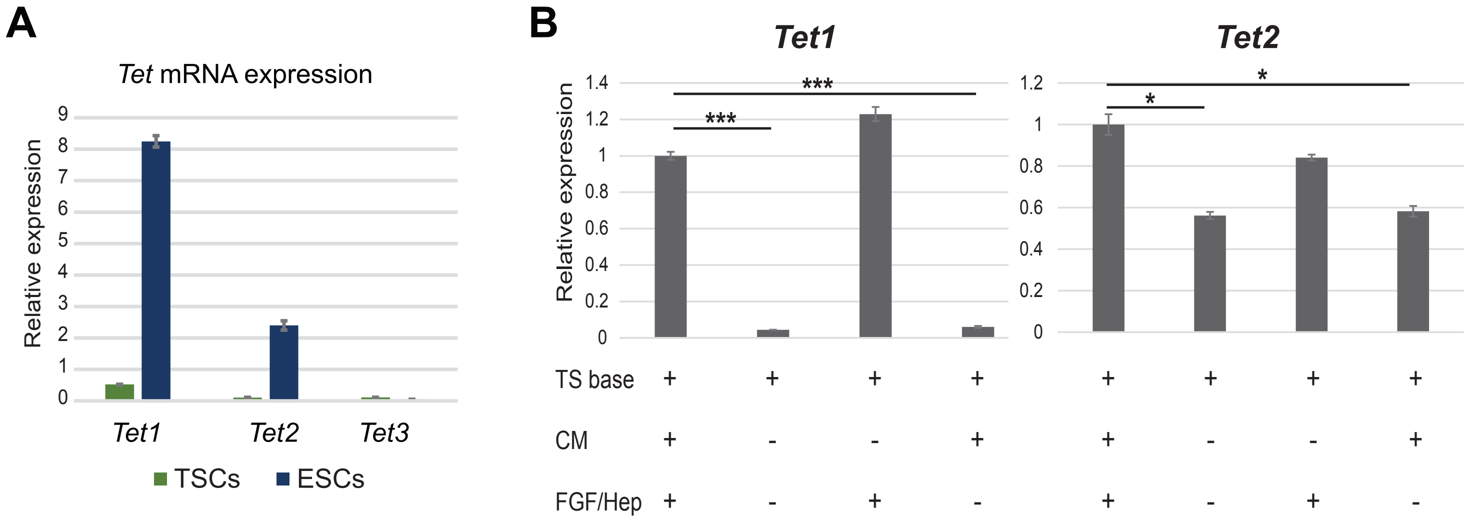

Figure S1. Regulation of *Tet* expression in TSCs. Related to Figure 1.
(*A*) RT-qPCR analysis of *Tet1,2,3* expression in TSCs and ESCs. Data are mean ± S.E.M. of n=3 independent replicates. (*B*) *Tet1* and *Tet2* mRNA expression in different trophoblast media composition, as indicated. Relative expression of each gene in standard TSC conditions (TS base + conditioned medium (CM) + FGF/Heparin) was set to 1. Data are mean ± S.E.M.; * $p<0.05$, ** $p<0.01$, *** $p<0.001$ (ANOVA with Holm-Bonferroni's post-hoc test); n=3 independent replicates.

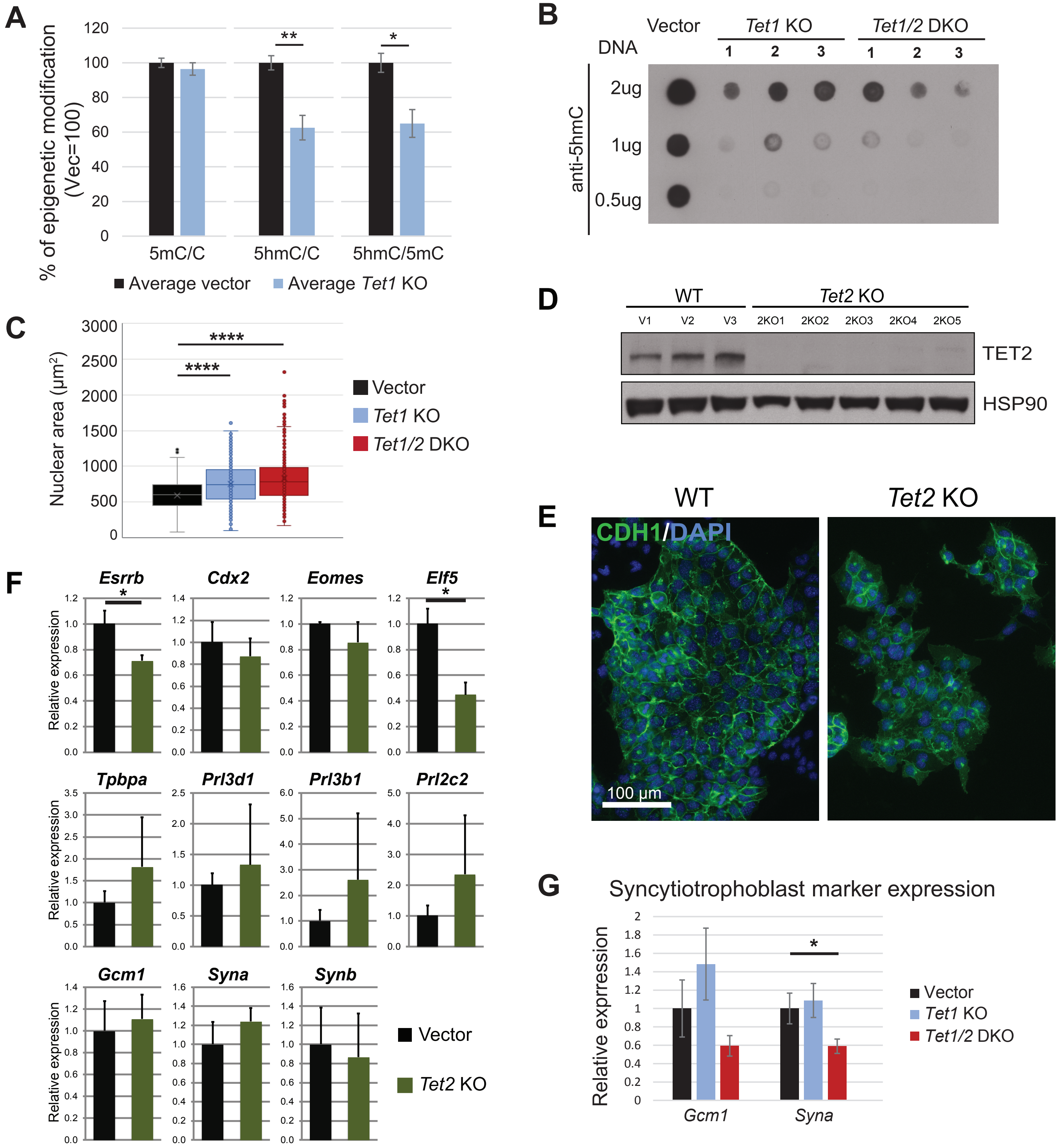

Figure S2. *Tet1/2* single and double KO clone generation and characterization. Related to Figure 1.
(*A*) Absolute quantification of 5-hydroxymethylcytosine (5hmC), 5-methylcytosine (5mC) and cytosine (C) by mass spectrometry in vector control (n=3 independent clones) and *Tet1* KO (n=3 independent clones) TSCs. Data are mean ± S.E.M.; * p<0.05, ** p<0.01 (unpaired, one-sided t-test). (*B*) DNA dot blot analysis for 5hmC levels in vector, *Tet1* KO and *Tet1/2* DKO clones. (*C*) Nuclear size of vector, *Tet1* KO and *Tet1/2* DKO cells determined by nuclear area measurements analysed by ImageJ. **** p<0.0001 (two-way ANOVA with Bonferroni's post-hoc test). Measurements are of >400 cells each. (*D*) Western blot for TET2 proving generation of *Tet2* null TSC clones. WT=empty vector control wild-type TSC clones. (*E*) Immunofluorescence staining for E-Cadherin (CDH1), demonstrating a loss of epithelial characterisics in *Tet2*-deficient TSCs similar to that observed in the absence of *Tet1*. (*F*) RT-qPCR analysis on vector control and *Tet2* KO TSCs for a cohort of trophoblast marker genes. Data are mean ± S.E.M.; * p<0.05 (ANOVA with Holm-Bonferroni's post-hoc test); n=4 clones as independent replicates each. (*G*) Expression of syncytiotrophoblast differentiation markers (*Gcm1*, *Syna*) in vector, *Tet1* KO and *Tet1/2* DKO clones grown in SCM for 3 days. Data are mean ± S.E.M.; * p<0.05 (ANOVA with Holm-Bonferroni's post-hoc test); n=4 clones as independent replicates each.

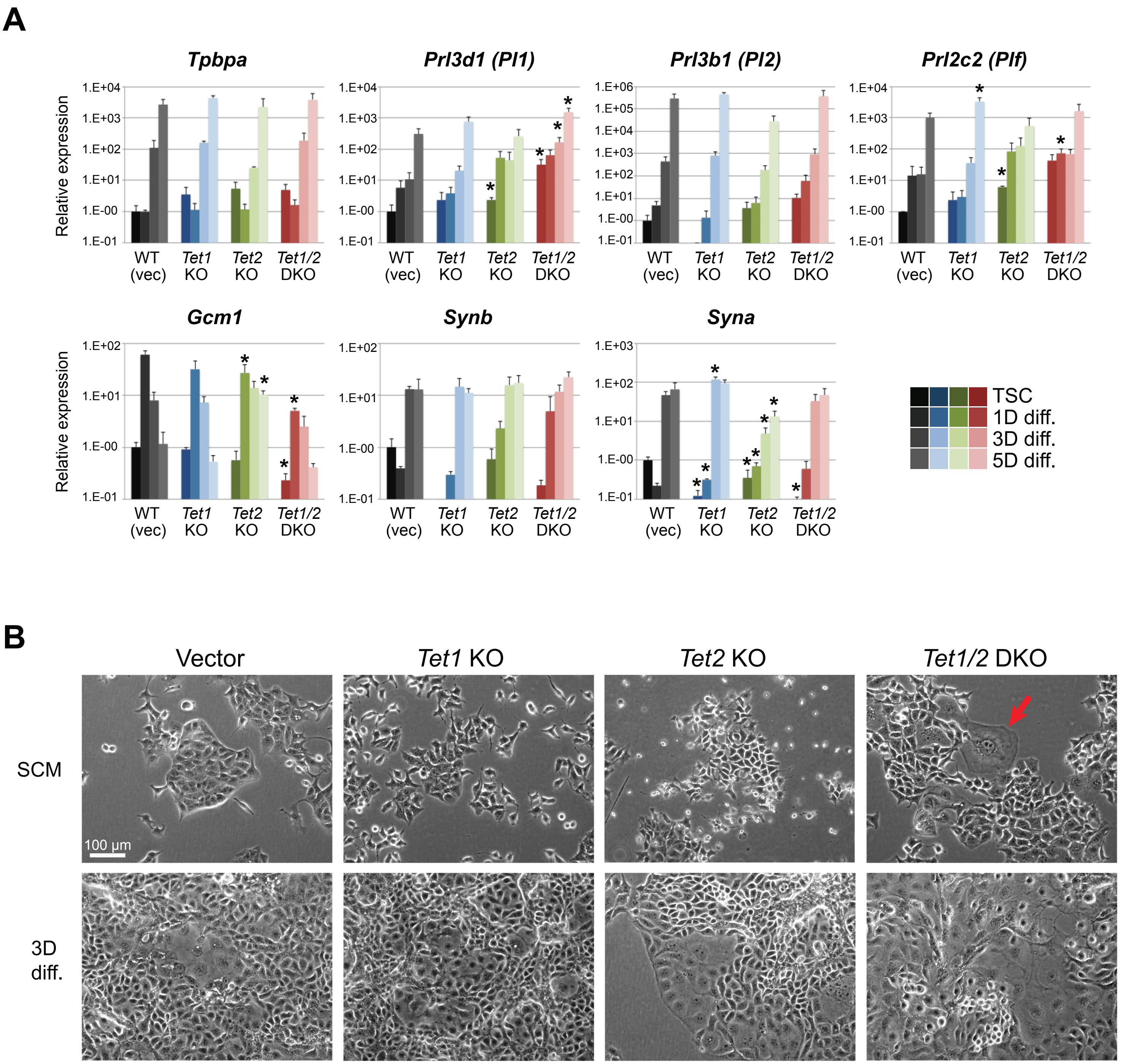

Figure S3. Differentiation characteristics of *Tet*-deficient TSCs. Related to Figure 1.
(*A*) RT-qPCR analysis across a TSC differentiation time course of up to 5 days (5D). Trophoblast differentiation marker genes were assessed. Data are mean ± S.E.M.; * $p<0.05$ (ANOVA with Holm-Bonferroni's post-hoc test); n≥3 clones as independent replicates for each genotype. Significance was calculated against the corresponding time point of vector control (wild-type) cells. (*B*) Phase contrast images of representative TSC clones of the indicated genotype grown in stem cell conditions (SCM) or after 3 days (3D) of differentiation. Note the increased number and size of trophoblast giant cells in particular in the *Tet1/2* DKO cells even in SCM conditions (arrow).

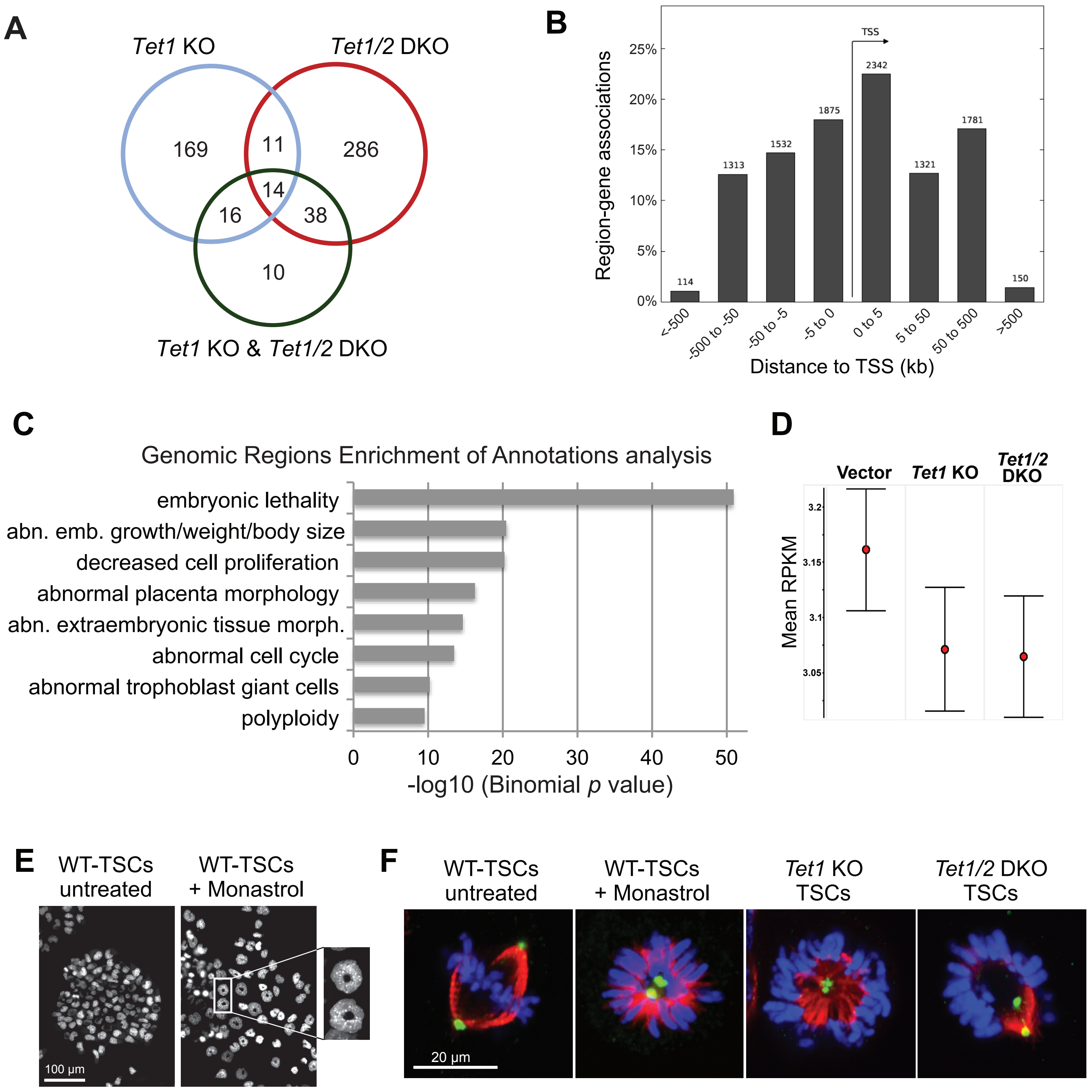

Figure S4. TET1/2 control cell adhesion and centrosome separation in TSCs. Related to Figure 2.
(*A*) Venn diagrams showing overlap of genes deregulated in *Tet1* KO (n=3) and *Tet1/2* DKO clones (n=3), and genes identified when *Tet1* KO and *Tet1/2* DKO cells are jointly compared against vector control TSCs. (*B*) Distribution of TET1 peaks in WT TSCs displayed in relation to transcriptional start site (TSS). (*C*) Genomic regions enrichment of annotations analysis (http://great.stanford.edu/public/html/) displaying some of the most significantly enriched terms. (*D*) Mean read count quantitation (RPKM) of genes bound by TET1 between -2kb and +500 bp of transcriptional start site. Whiskers are confidence interval (S.E.M.). (*E*) Wild-type (WT) TSCs treated with the Kinesin EG5 inhibitor Monastrol acquire ring-shaped nuclei akin to those observed in *Tet1*- and *Tet1/2*-null TSCs. (*F*) Immunofluorescence staining for α-Tubulin (red) and γ-Tubulin (green) in WT-TSCs, TSCs treated with 100µM Monastrol for 20 hr, *Tet1* KO and *Tet1/2* DKO trophoblast cells. DAPI staining (blue) was used to identify live nuclei. Images were taken with oil-immersion objective by confocal microscopy.

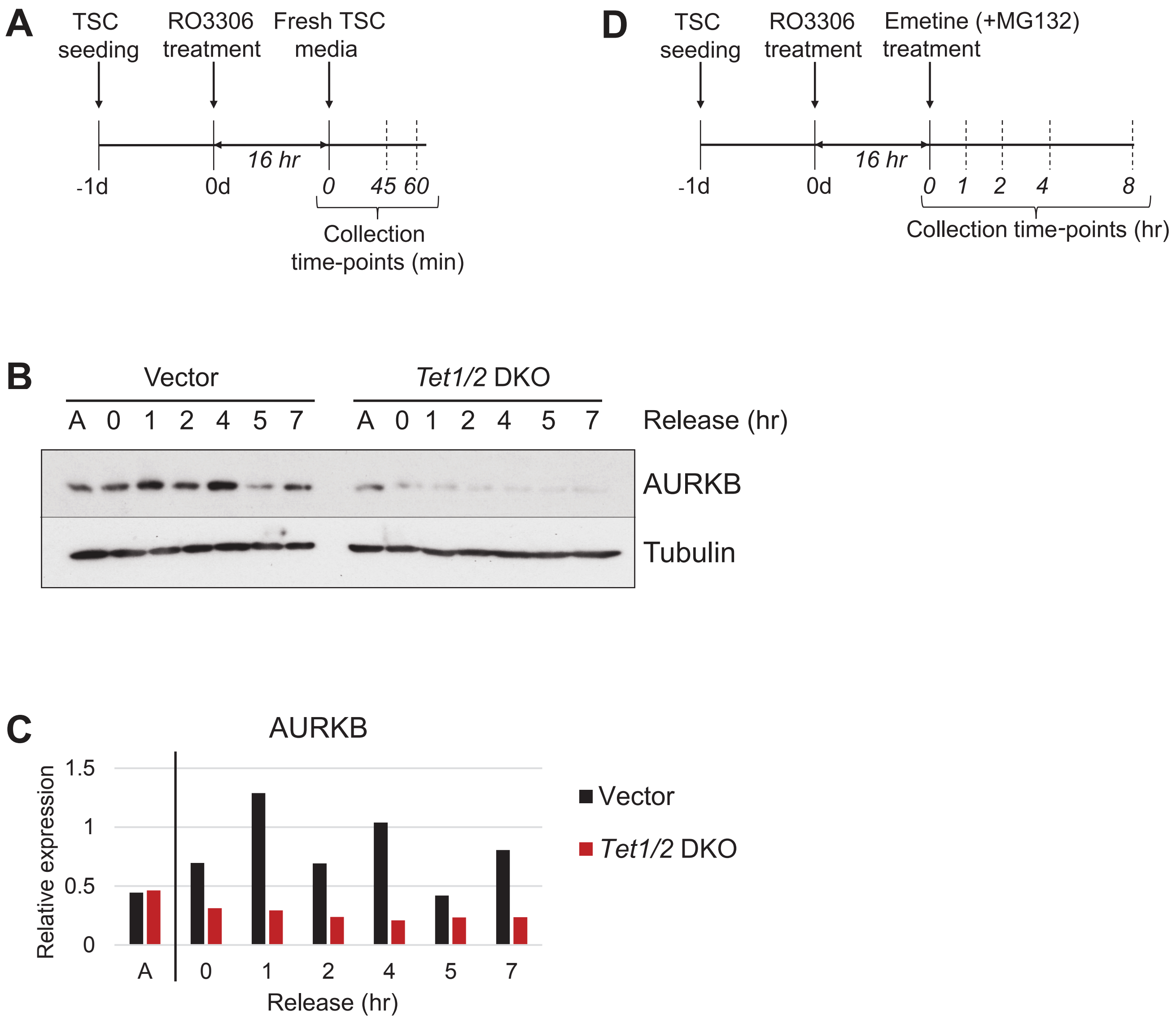

Figure S5. TET1/2 are required for normal G2/M progression. Related to Figure 4 and Figure 5.
(*A*) Schematic outlining experimental protocol: cells were seeded and left to adhere to the dish for 1 day. RO3306, a CDK1 inhibitor was added to the TSC complete media for 16 hr, arresting the cells at G2/M phase. The G2/M arrested cells were released with fresh TSC media and they were collected for analysis at the timepoints indicated. (*B*) Lysates collected at the indicated time-points were analysed by Western blot. (*C*) Quantification of Western blot shown in (*B*) after normalised to Tubulin. (*D*) Schematic outlining experimental protocol: Emetine, a translation inhibitor, was added to G2/M arrested vector and *Tet1/2* DKO cells for the times indicated to study Cyclin B1 stability.

Table S3. Primer sets and gRNA sequences used in this study. Related to Figures 1, 2 and 3.

Target gene	Primer	Sequence
Tet1 gRNAa (exon 5)	F	5'- CACCGGCTGCTGTCAGGGAGCTCA -3'
	R	5'- AAACTGAGCTCCCTGACAGCAGCC -3'
Tet1 gRNAb (exon 4)	F	5'- CACCGAGAATGCAGGCGGCATGAC -3'
	R	5'- AAACGTCATGCCGCCTGCATTCTC -3'
Tet1 gRNAc (exon 2)	F	5'- CACCGTAAACCCTTGTGGGACGGC -3'
	R	5'- AAACGCCGTCCCACAAGGGTTTAC -3'
Tet2 gRNAa (exon 3)	F	5'- CACCGAAAGTGCCAACAGATATCC -3'
	R	5'- AAACGGATATCTGTTGGCACTTTC -3'
Tet2 gRNAb (exon 3)	F	5'- CACCGGAGTTCGGTTGCTTCGGTT -3'
	R	5'- AAACAACCGAAGCAACCGAACTCC -3'
Tet2 gRNAc (exon 3)	F	5'- CACCGTCTGTAAGTTGCTCGAATT -3'
	R	5'- AAACAATTCGAGCAACTTACAGAC -3'
Tet1	F	5'-GAGCCTGTTCCTCGATGTGG-3'
	R	5'-CAAACCCACCTGAGGCTGTT-3'
Tet2	F	5'-AACCTGGCTACTGTCATTGCTCCA-3'
	R	5'-ATGTTCTGCTGGTCTCTGTGGGAA-3'
Tet3	F	5'-TCCGGATTGAGAAGGTCATC-3'
	R	5'-CCAGGCCAGGATCAAGATAA-3'
Cdx2	F	5'-AGTGAGCTGGCTGCCACACT-3'
	R	5'-GCTGCTGCTGCTTCTTCTTGA-3'
Esrrb	F	5'-AGTACAAGCGACGGCTGG-3'
	R	5'-CCTAGTAGATTCGAGACGATCTTAGTCA-3'
Elf5	F	5'-ATTCGCTCGCAAGGTTACTCC-3'
	R	5'-GGATGCCACAGTTCTCTTCAGG-3'
Tpbpa	F	5'-ACTGGAGTGCCCAGCACAGC-3'
	R	5'-GCAGTTCAGCATCCAACTGCG-3'
Pl1	F	5'-TTATCTTGGCCGCAGATGTGT-3'
	R	5'-GGAGTATGGATGGAAGCAGTATGAC-3'
Plf	F	5'-AACGCAGTCCGGAACGGGG-3'
	R	5'-TGTCTAGGCAGCTGATCATGCCA-3'
Syna	F	5'-CCTCACCTCCCAGGCCCCTC-3'
	R	5'-GGCAGGGAGTTTGCCCACGA-3'
Gcm1	F	5'-GCTCCACAGAGGAAGGCCGC-3'
	R	5'-GTTGGTGACCGGGAAGCCGC-3'
Dynein	F	5'-GACCTCAGGCTCAGACGAAGAC-3'
	R	5'-AAGACGCTCATGGCATCACA-3'

Sdha	F	5’-TGGTGAGAACAAGAAGGCATCA-3’
	R	5’-CGCCTACAACCACAGCATCA-3’
Cdk1	F	5’-GTCCGTCGTAACCTGTTGAG-3’
	R	5’-TGACTATATTTGGATGTCGAAG-3’
Cdk2	F	5’-TCTGCTCTCACGGGCATTC-3’
	R	5’-AGCTGGAACAGATAGCTCTTGATGA-3’
Cyclin B1	F	5’-GCGAAGAGCTACAGGCAAGA-3’
	R	5’-CACCTCTGGTTCACACACAG-3’
Cyclin D1	F	5’-AGGAGCAGAAGTGCGAAGAG-3’
	R	5’- CACAACTTCTCGGCAGTCAAG-3’
Cyclin E1	F	5’-TGTTTTTGCAAGACCCAGATGA-3’
	R	5’-GGCTGACTGCTATCCTCGCT-3’
Cyclin E2	F	5’- GGAACCACAGATGAGGTC-3’
	R	5’- CGTAAGCAAACTCTTGGAG-3’
Vim	F	5’-ACTGCAGGAGCTGAATGACC-3’
	R	5’-CGCATCTCCTCCTCGTACAG-3’
Cdh2	F	5’-GTCTGTGGAGGCTTCTGGT-3’
	R	5’-GTCCTCGTCCACCTTGAAA-3’
Zeb2	F	5’-GAGGAAAGAGATGGCCACG-3’
	R	5’-GTCAGCAGTTGGGCAAAAG-3’
Snai2	F	5’-CCTTTCTCTTGCCCTCACTG-3’
	R	5’-ACAGCAGCCAGACTCCTCAT-3’
Snai1	F	5’-CTTGTGTCTGCACGACCTGT-3’
	R	5’-CTTCACATCCGAGTGGGTTT-3’
Cdh1 BS	1F	5’- CTACACGACGCTCTTCCGATCTTTGGGGAAAATAGTTTATTGTGAAATAGAT -3’
Cdh1 BS	1R	5’- TGCTGAACCGCTCTTCCGATCTNNNNNNNNAAATACTTCTCCCACCTAAACCACC -3’
Cdh1 BS	2F	5’- CTACACGACGCTCTTCCGATCTAATTTTTGGAGGGTGGATATTATTTTAGTT -3’
Cdh1 BS	2R	5’- TGCTGAACCGCTCTTCCGATCTNNNNNNNNCCTTAACCTCCCAAAAACTAATCAC -3’
